# Elemental Mercury Spill in School Bus and Residence — North Carolina, 2013

**Published:** 2014-02-14

**Authors:** Ricky Langley, Anne Hirsch, Jesse McDaniel, Valerie Lott, Mina Shehee, Samantha Migit, Anna Rouse Dulaney, Jose Negron, Aaron Fleischauer

**Affiliations:** 1 North Carolina Division of Public Health; 2 Iredell County Health Department; 3 Carolinas Poison Center; 4 Environmental Protection Agency; 5 Career Epidemiology Field Officer Program, CDC

On September 16, 2013, the North Carolina Division of Public Health was notified of an elemental (metallic and liquid) mercury spill on a school bus. An elementary student boarded the bus with approximately 1 pound (454 g) of elemental mercury contained in a film canister, which the student had taken from an adult relative who had found it in a neighbor’s shed. The canister was handled by several students before the contents spilled on the bus floor. Ten passengers aboard the bus were exposed, including eight students and two staff members. Although elemental mercury is not readily absorbed from skin contact or ingestion, it does vaporize at room temperatures and inhalation of the vapor can be harmful. The bus driver promptly notified school officials. Firefighters and a local hazardous materials team directed decontamination procedures (i.e., changing clothes and washing hands and shoes) for the 10 exposed passengers. The bus was immediately taken out of service and sent for disposal because of its age and the cost of decontamination.

An Environmental Protection Agency (EPA) response team used a mercury vapor analyzer to determine mercury vapor levels at the residence from which the mercury was taken and at the three schools where the children were dropped off. The residence had mercury levels of 673 *μ*g/m^3^, which is higher than the Agency for Toxic Substances and Disease Registry’s recommended levels for residential cleanup (1 *μ*g/m^3^) and evacuation (≥10 *μ*g/m^3^) (1). Over a 10-day period, the EPA response team remediated the contaminated residence through ventilation, removal of free mercury and mercury-contaminated items (e.g., furniture, carpet, bedding, and clothing), cleaning of surfaces with a mercury binding solution, and heating of the residence. EPA, Iredell County Emergency Management, Iredell County Health Department, American Red Cross-Greater Carolinas Chapter, and Iredell County Department of Social Services collaborated to assist the family with shelter, food, clothing, transportation, and medical needs during the response and recovery phases. Testing with a mercury vapor analyzer at the three schools potentially affected did not indicate contamination, with the exception of several pieces of carpet removed from one classroom.

To quantify human exposure and assess symptoms, the Iredell County Health Department administered a mercury exposure questionnaire to 23 persons, including the 10 exposed passengers aboard the school bus, seven family members who lived at the contaminated residence, two family members who had visited the residence 2 days before the exposure on the bus, and four firefighters. The North Carolina State Laboratory of Public Health performed blood mercury testing on 12 of the 23 persons.

Two students and three family members reported acute symptoms on the day of the exposure, including headache, cough, numbness or tingling in hands, and difficulty breathing. The student who brought the mercury aboard the bus and five family members, including two adults, had elevated blood mercury levels, ranging from 134 *μ*g/L to >200 *μ*g/L. A blood mercury concentration of ≥50 *μ*g/L is considered the threshold for symptoms of toxicity after an acute high level exposure (2). Two children who had symptoms and blood mercury levels >200 *μ*g/L received a 19-day course of dimercaptosuccinic acid chelation therapy (2). Two other children with elevated blood mercury levels but no symptoms were followed every 2 weeks with urine testing until levels normalized. The two adults were referred to their physician for follow-up.

Through this investigation, six persons with blood mercury levels exceeding human health risk thresholds were identified. Two of these persons required chelation therapy. To prevent mercury spills in schools and residences, continued efforts should be made to educate school children, school employees, and the public about the dangers of possessing and handling mercury (3). Prompt actions by trained school personnel were critical in bringing this incident to the attention of authorities and avoiding further contamination.
